# Determinants of birthweight in rural Sri Lanka; a cohort study

**DOI:** 10.1186/s12887-022-03830-0

**Published:** 2023-01-23

**Authors:** Premarathnage Dhammika Narendra Premarathne Banda, Gayani Shashikala Amarasinghe, Suneth Buddhika Agampodi

**Affiliations:** 1 Medical Officer of Health Office, Palagala, Sri Lanka; 2 Department of Community Medicine, Faculty of Medicine and Allied Sciences, Rajarata University of Sri Lanka, Saliyapura, Sri Lanka; 3 Department of Community Medicine, Faculty of Medicine and Allied Sciences, Rajarata University of Sri Lanka, Saiyapura, Sri Lanka

**Keywords:** Birthweight, Cohort, Sri Lanka; Pregnancy, Violence, Domestic cohesion

## Abstract

**Background:**

Identifying determinants of birthweight among disadvantaged communities is critical to further reducing the inequitable burden of perinatal health issues in low-and-middle income settings. Therefore, we adopted a bio-psycho-social approach to identify the determinants of birthweight in a mother-infant cohort from a rural setting in Sri Lanka, a lower-middle-income country.

**Methods:**

All third-trimester pregnant women with a singleton pregnancy registered for the national antenatal care programme at Ipalogama health division in 2017 were invited for a prospective cohort study. Data was collected using a self-completed questionnaire and data extraction from health records. The mother-infant cohort was followed up until one month after delivery. A principal component analysis was performed using economic, social, and psychological variables, and two composite variables were achieved. Care from husband and household members, perceived wellbeing, frequency of abuse, and affect during the third trimester strongly loaded to the variable 'psychosocial wellbeing'. Monthly income, husband's education level, and use of biomass fuel strongly loaded to the variable 'socioeconomic status'. Hierarchical logistic regression was used to predict factors associated with birthweight. Maternal age, parity, baby's sex, and gestational period at pregnancy registration were entered at the first step. BMI, psychosocial wellbeing, socioeconomic status, hypertensive disorders, and gestational/chronic diabetes were entered at step two. Preterm birth was entered at step three.

**Results:**

532 women were recruited, and 495 were retained at the postpartum follow-up. 421 (74.8%) had reported being abused at least once during the preceding month. Birthweight was approximately normally distributed (mean 2912 g, SD 456.6 g). Low birthweight was present in 72 (14.6%, 95% CI 11.7,17.9), and 46 (9.3%, 95% CI 7.0,12.1) had birthweights > 3500 g. The regression model explained 13.2% of the variance in birthweight. Preterm birth, maternal BMI, and mid-pregnancy psychosocial wellbeing could explain 6.9%(*p* < 0.001), 3.9(*p* < 0.001), and 1.2%(*p* = 0.02) of unique variance, respectively.

**Conclusions:**

In a setting where a large proportion of pregnant women suffer 'abuse' in their homes, psychosocial wellbeing during pregnancy was an important determinant of birthweight of babies. Expanding routine maternal care services, especially at the primary care level, to cater to the psychosocial issues of pregnant women would help reduce inequities in perinatal health.

## Background

Birthweight is the first weight of the fetus obtained after birth. Globally, 15% to 20% of babies are born with low birthweight (less than 2500 g), and the issue disproportionately affects babies from low and middle-income countries [1]. In either setting, marginalized and disadvantaged population subgroups show a higher prevalence of low birthweight and its associated complications, including mortality and ill health, demonstrating a cycle of health inequity [2–4].

Birthweight is unique since it reflects the cumulative impact of numerous factors on the baby's health. These include quality of maternal care services[5, 6], preconceptional and antenatal nutritional status of the mother [6, 7], maternal comorbidities [8], socioeconomic conditions [4, 6], genetics and epigenetics [9]. Intermediary social determinants of health such as the living and working environment [8, 10–14], food security [15], social capital and experiencing violence [11, 16, 17], and mental health of the mother [18, 19] can affect the birthweight of their babies. Preterm deliveries, either spontaneous or provider-initiated (due to medical, obstetric, or other reasons), also are a major determinant of lower birthweight [6, 8, 18, 19]. Birthweight can predict many short- and long-term adverse health outcomes in children. Neonates with low birthweight are 20 times more likely to die during infancy than their counterparts [14]. Being very low birthweight can affect children's cognitive function and increase the risk for diseases such as asthma and chronic kidney disease [20–22]. An association has been shown between birthweight and non-communicable diseases such as hypertension and type 2 diabetes [23, 24]. Birthweights at the higher end of the spectrum are associated with the risk of obesity and cancers [25].

Global nutrition targets aim for a 30% reduction in the global incidence of low birthweight by 2025 [26]. However, progress toward this goal is slower than expected [27]. Similar to the rest of the underperforming nutritional indicators albeit having a robust public health system performing well in other aspects of maternal and child health, low birthweight prevalence is comparatively high in Sri Lanka [28]. Many interventions have already been implemented to control the low birthweight incidence, including almost all evidence-based interventions recommended by the WHO in the policy brief on the world health assembly's low birthweight targets. However, similar to the global situation, little progress has been achieved over the past decades [29]. Shifts in the epidemiology of diseases in pregnancy, such as gestational diabetes, may further complicate the picture, increasing the number of births with higher birthweights [30].

Further improvements in birthweight require detailed assessments of contributory factors, especially those often overshadowed by prominent nutritional issues. Going by the overarching principle of sustainable development goals, "leaving no one behind", addressing issues concentrated in specific sub-population groups is also important. Therefore, we adopted a bio-psycho-social approach to study the determinants of birthweight in a mother-infant cohort from a rural setting in a middle-income country.

## Methods

### Study design

A prospective cohort study was carried out in Ipalogama Medical Officer of Health (MOH) area, Sri Lanka, from January 2017 to June 2018. Ipalogama is one of the 22 health divisions (MOH areas) in the Anuradhapura district. It has a population of 44,218, and 682 live births were reported to the reproductive health information system in 2017 (86% of estimated births). Field antenatal care is provided to pregnant women through antenatal clinics and domiciliary visits under the national maternal care package, and 90% of the estimated pregnancies at Ipalogama had registered for antenatal care. Over 99.9% of deliveries occurred in hospitals [31]. Women receive domiciliary care during the postpartum, and a postpartum clinic is scheduled at four weeks.

All pregnant women with a period of gestation (PoG) of 28 weeks or more who were registered for antenatal care in the Ipalogama MOH area during 2017 were invited to participate. Pregnancies with multiple gestations were excluded. Recruitment was done consecutively at antenatal clinics.

**Fig. 1:**
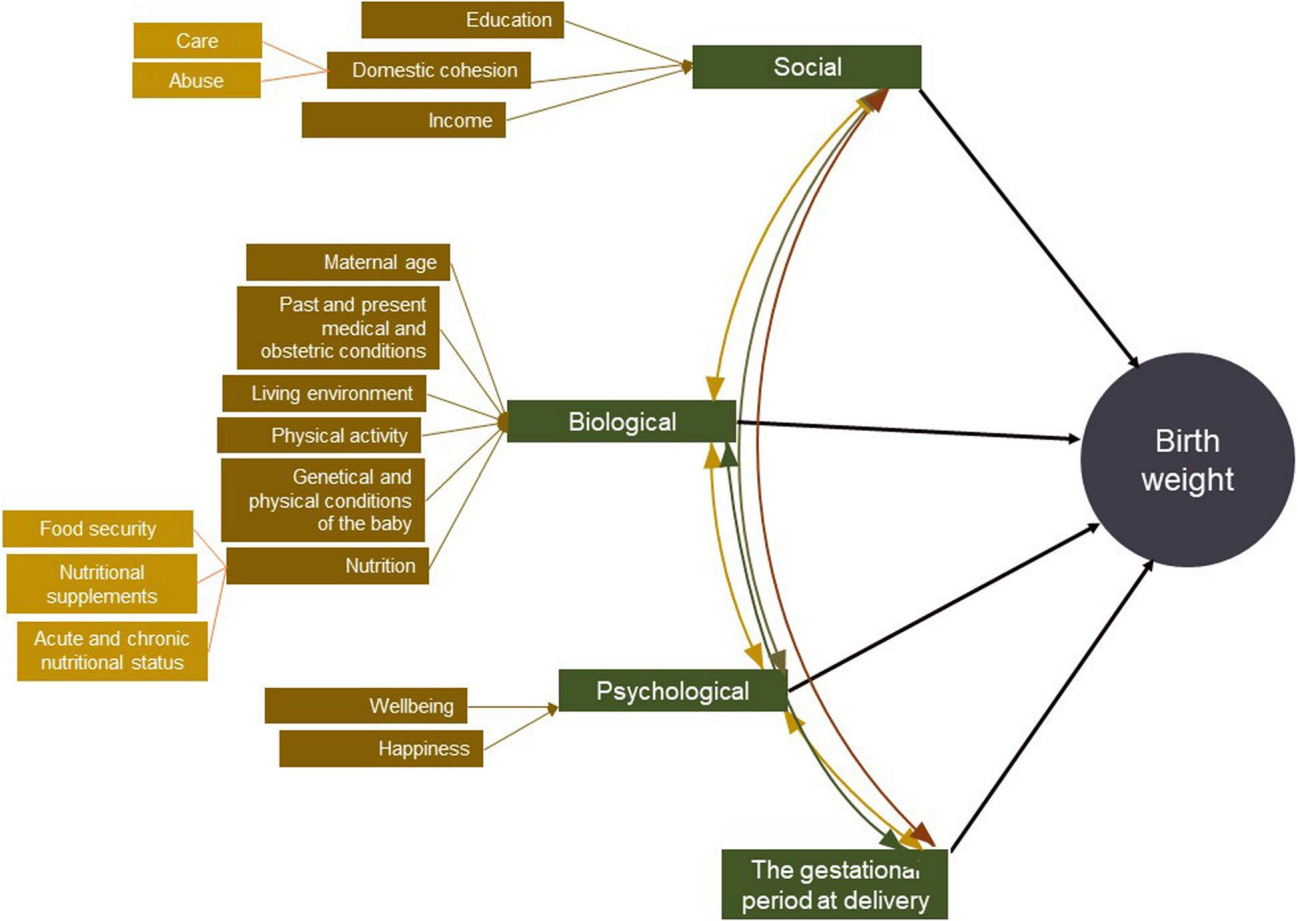
Conceptual framework used for the study

The conceptual framework for the study is shown in Fig. 1. Baseline data was collected using a self-completed questionnaire and data extraction from the pregnancy record. Data regarding the participant's and partner's demographic characteristics, obstetric and medical history, anthropometric measurements at the time of pregnancy registration, hemoglobin and plasma glucose values at the registration, and around 28 weeks of gestation were extracted from the pregnancy record of the mother. Whether the symphysio-funadal height (SFH) is compatible with the PoG and if the pregnancy weight gain is according to the national recommendations were checked from the pregnancy record and marked in the data extraction sheet. To improve the data quality, filling the pregnancy record by the field health staff was standardized and randomly cross-checked by the principal investigator.

The self-completed questionnaire was used to collect data on monthly family income, environmental conditions, physical activity patterns, food security, and compliance with nutritional supplementations (self-reported). To assess domestic cohesion, participants were asked to rate (on a scale of 1 to 10) the care they received from their husbands and other household members. Whether the participants were being 'abused' at home was assessed by asking about the frequency (during the month prior to baseline assessment) their husband or another household member had verbally, physically, or sexually done things that made them feel unhappy/sad. A pictogram of six faces was used to rate their general affect during the preceding week. Perceived wellbeing during the preceding month was rated on a scale of 1 to 10. The first follow-up was scheduled for one month after the baseline data collection.

### Outcome variables

According to national guidelines, birthweight should be measured in all neonates born at hospitals as soon as possible (ideally within two hours) using calibrated equipment, and this should be recorded in the child health development record (CHDR) of the baby [32]. During the postpartum clinic (scheduled around one month after birth), this entry in the babies' CHDR was extracted along with the period of gestation (PoG) at birth and mode of delivery.

### Statistical analysis

Data analysis was done using IBM SPSS version 22. T-test and two-way ANOVA were used to see the difference in mean birthweight among babies whose mothers were categorized by different characteristics.

Since psychosocial and socioeconomic status variables can be correlated, a principle component analysis was used to create composite variables. Kaiser–Meyer–Olkin's measure of sampling adequacy was 0.8, and Bartlett's test of sphericity Approx. Chi-Square was 1245.3, *P* < *0.001* supporting the factorability of the correlation matrix. A two-factor solution was reached. With Catell's scree test, both factors were decided to be kept. This decision was further supported by parallel analysis, which showed both components exceeding the corresponding criterion value for a randomly generated data matrix of the same size. The oblimin rotation provided a simple structure with both components showing several strong loadings and all variables loading substantially on only one component. Factor scores were recorded and used in further analysis.

Hierarchical logistic regression was performed to identify factors associated with birthweight. Preliminary analyses were conducted to ensure no violation of normality, linearity, multicollinearity, and homoscedasticity assumptions. Parity (primy/ multi), sex of the baby (Male/Female), Maternal age (years), and PoG at pregnancy registration (weeks) were entered in step 1. In step 2, whether having chronic/gestational diabetes, chronic or pregnancy-induced hypertension, BMI at the time of pregnancy registration, factor scores for psychosocial wellbeing, and Socioeconomic status were entered. In the third step, whether preterm or not was entered. Value for part correlation was squared to identify the percentage of unique variance explained by the predictor variables in the model.

## Results

Out of 532 pregnant women enrolled, the baby's birthweight data was available in 497 (Lost to follow-up 6.5%). Participants were between 16 to 44 years of age. The mean age was 28.5 years (SD 5.6). The median monthly family income was 205.6 USD (Minimum 19.7 USD, Maximum 2951 USD). The majority of the participants were ethnic Sinhalese (*n* = 423, 80.4%), were unemployed (*n* = 437, 82.1%), and had completed education at least up to grade 11 (*n* = 298, 57.2). One-fifth were from farming families (*n* = 114, 21.5%). Of the participants, 18 (3.4%) reported that during the month prior to the baseline assessment, their husband or another family member had verbally/physically, or sexually done things that made them feel unhappy/sad on a daily basis. Another 25 (4.8%) reported they faced such incidents several times a week, while 75 (14.3%) and 263 (50.3%) had similar "abuse" several times a month and less often, respectively. Only 142 (25.2%) reported not experiencing such violence.

The gestation period at the time of birth varied between 29 weeks plus one day to 42 weeks and four days, with a mean of 38 weeks and six days. There was an entry with 43 weeks and two days PoG with an ultrasound confirmation. Of all births, 61(11.5%, 95% CI 9.06,14.41) were preterm ( born at 36 weeks plus six days or earlier PoG).

**Fig. 2:**
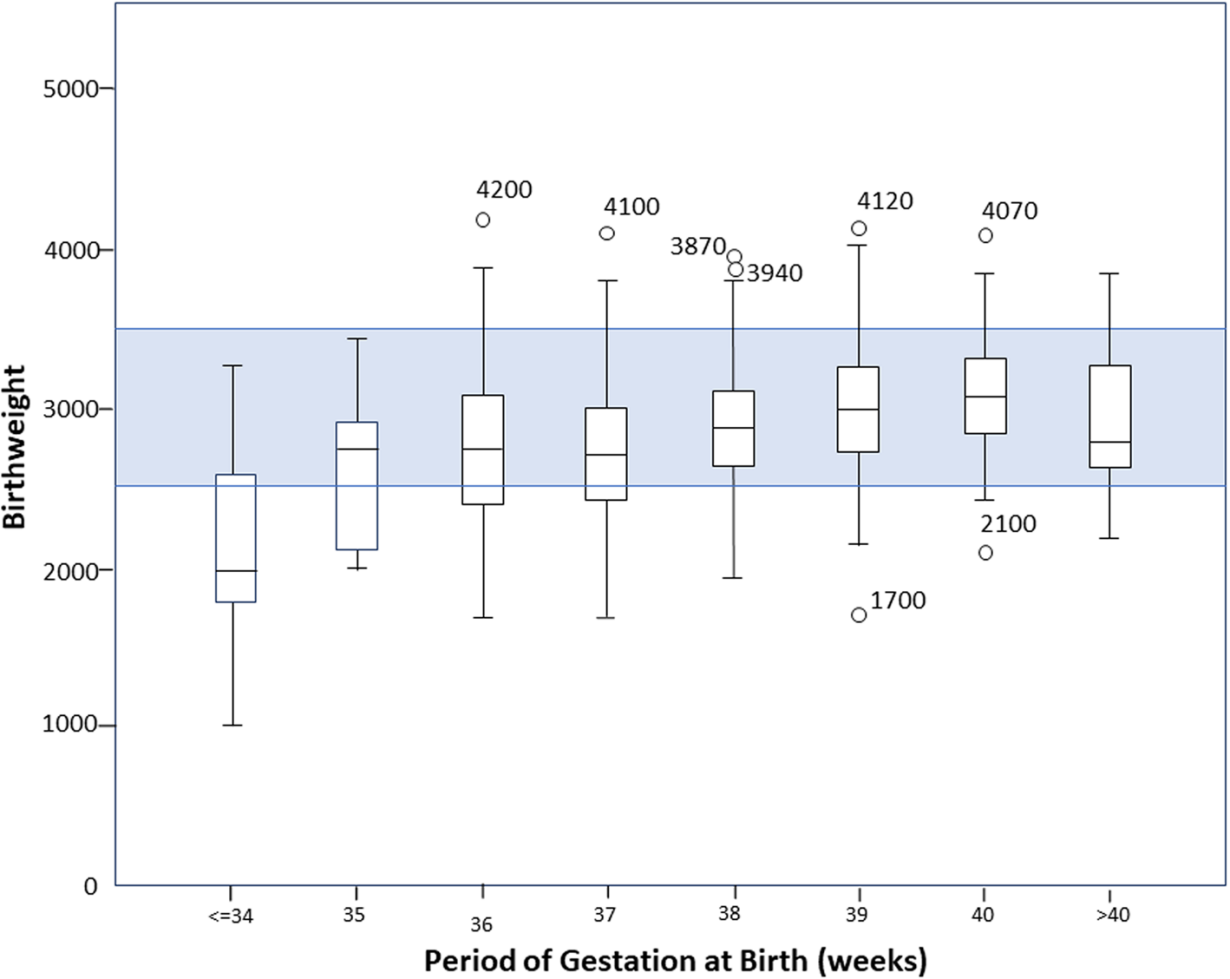
Distribution of Birth Weight According to the Period of Gestation at Birth

Birthweight was approximately normally distributed (Skewedness = -0.21) and ranged from 1020 to 4200 g. The mean birthweight was 2912 g (Standard deviation 456.6 g). Of the babies, 72 (14.6%, 95% CI 11.66,17.85) had low birthweight, while 46 (9.3%, 95% CI 7.01,12.13) had higher birthweight (≥ 3500 g). The distribution of birthweight according to the PoG at birth is shown in Fig. 2.

### Bivariate analysis for factors associated with birthweight

In the bivariate analysis (Table 1), female babies (*t* = 2.2, *P 0.03*) and babies born to teenage mothers (less than 20 years of age) (*t* = -1.70, *P 0.09*) were comparatively lighter. Babies of mothers with low educational level (*t* = -1.7, *P 0.09*), mothers who had a history of giving birth to low birthweight babies (*t* = -6.7, *P 0.01*), gestational diabetes mellitus or diabetes during pregnancy (*t* = 2.5, *P 0.01*) and who miss calcium supplementation (t = -2.306*, P 0.022*) had lower mean birthweight compared to their counterparts. All the factors studied under domestic cohesion and psychological factors (Table 1) were significantly associated with the birthweight of the baby.

**Table 1:** Factors associated with birth weight among 530 pregnant women from Ipalogama, Sri Lanka

		**N**	**Mean birthweight**	**Std. Deviation**	**t/f (*P)***
**Demographic factors**					
Has completed education up to grade 11	Yes	273	2949.6	461.5	2.10(0.04)
	No	213	2862.0	456.0	
Husband has completed education up to grade 11	Yes	269	2954.8	444.7	2.40(0.02)
	No	216	2853.5	474.2	
Teenage mother	Yes	23	2751.5	554.3	-1.70(0.09)
	No	472	2915.9	452.3	
Monthly family income (Rs.10000)	> 10,000	449	2914.6	462.6	0.70(0.46)
	≤ 10,000	46	2862.2	417.3	
**Obstetric history of the mother**					
Primy gravida	Yes	134	2834.6	396.8	-2.50(0.01)
	No	358	2940.2	477.2	
Having a previous baby with a low birth weight	Yes	79	2638.9	464.3	-6.70 (< 0.01)
	No	268	3026.2	449.1	
**Characteristics of the baby**					
Sex of the baby	Male	251	2950.4	483.7	2.20(0.03)
	Female	243	2861.8	425.3	
**Medical conditions of the mother**					
Chronic/gestational diabetes	Yes	28	3115.9	556.5	2.50(0.01)
	No	467	2896.5	449.6	
Chronic/ pregnancy-induced hypertension	No	478	2913.8	450.0	0.40(0.67)
	Yes	15	2837.0	667.3	
**Nutritional status of the mother at pregnancy registration**					
Stunting in mother	Yes	25	2608.6	506.9	-3.40(0.00)
	No	471	2925.8	450.4	
Maternal BMI	< 18.5	82	2823.7	418.3	7.77(0.00)
	18.5–22.9	166	2820.2	435.0	
	23–24.9	68	2943.1	451.6	
	> = 25	159	3038.9	471.8	
**Food and nutritional supplements**					
Number of meals missed per week	≥ 1	129	2843.3	433.622	-1.98(0.05)
	none	359	2935.8	463.531	
Shares her allocated 'Triposha' supplement with family	No	95	2834.5	473.4	-1.85 (0.06)
	Yes	400	2935.3	477.2	
Missed doses of iron supplement per week	≥ 2.0	81	2892.8	395.2	-0.50(0.63)
	< 2.0	406	2919.3	466.6	
Missed doses of calcium supplements per week	≥ 2.0	131	2837.5	419.2	-2.30(0.02)
	< 2.0	355	2943.8	461.8	
**Environmental factors**					
Exposure to smoke of biomass fuel at least once a day	Yes	326	2895.8	450.5	-1.10(0.28)
	No	169	2943.2	468.0	
Use of biomass fuel at home	Yes	383	2886.3	444.3	-2.40(0.02)
	No	109	3003.7	489.0	
Exposure to tobacco smoke	Yes	68	2893.0	462.6	-0.40(0.71)
	No	423	2915.3	457.1	
**Physical activity pattern**					
Period of standing (per day)	≥ 1 h	171	2911.6	478.4	-0.30(0.77)
	< 1 h	307	2924.3	445.1	
Period of engagement in strenuous work (per day)	< 1 h	339	2918.9	457.1	0.10 (0.92)
	≥ 1 h	129	2913.8	454.3	
**Domestic cohesion**					
Perceived care from husband	Optimal	229	2957.9	475.0	2.20(0.03)
	Suboptimal	261	2866.8	441.4	
Perceived care from family members	Optimal	231	2947.1	454.9	1.70(0.09)
	Suboptimal	258	2877.2	461.5	
Frequency of being verbally/ physically/ sexually abused	At least a few times a month	110	2824.0	482.9	2.5(0.03)
	Occasionally / never	377	2934.5	446.4	
**Psychological**					
General affect over one week	Unhappy	83	2816.3	474.2	-2.20(0.03)
	Happy	405	2934.3	450.5	
Perceived wellbeing	Optimal	195	2972.6	451.5	2.50(0.01)
	Suboptimal	294	2868.3	460.9	

Fasting plasma glucose level at pregnancy registration was weakly correlated with birthweight (*r* = 0.19, *P 0.02*, *n* = 159). Fasting plasma glucose at 28 weeks of gestation (*r* = -0.06, *P 0.57*, *n* = 94), plasma glucose two hours following oral glucose tolerance test at the pregnancy registration (*r* = 0.07, *P 0.21*, *n* = 322), and 28 weeks of gestation (*r* = 0.05, *P 0.57*, *n* = 159), and postprandial blood sugar values (PPBS) at pregnancy registration (*r* = -0.01, *P 0.84*, *n* = 191) and 28 weeks of gestation (*r* = 0.07, *P 0.36*, *n* = 172) were not correlated with birthweight.

Birthweight had a moderate correlation with weight (*r* = 0.3, *P* < *0.001*) and a small correlation with height (*r* = 0.23, *P* < *0.001*) and BMI (*r* = 0.22, *P* < *0.001*) of the mother measured at the time of registration to antenatal care. There was no correlation between the baby's birthweight and maternal hemoglobin values (*r* = -0.4, *P 0.38* for hemoglobin at early and *r* = 0.02, *P 0.74* at mid-pregnancy) nor paternal height (*r* = 0.03, *P 0.47*).

Total weight gain during pregnancy was weakly correlated with babies' birthweight (r = 0.094, *P 0.038*). However, compatibility of SFH with the PoG (t -0.1, *P 0.92*) and gaining weight during pregnancy according to recommended ranges (t = 1.4*, P 0.16*) were not associated with the birthweight.

#### Psychosocial wellbeing and socioeconomic status

The factor analysis yielded a two-factor solution explaining 56.5% of the variance. The factor loading components were identified as psychosocial wellbeing (care from husband, care from household members, perceived wellbeing, frequency of abuse, and affect strongly loading) and socioeconomic status (monthly family income, education level of husband, and use of biomass fuel strongly loading) (Table 2). Psychosocial wellbeing and socioeconomic status explain 39.1% and 17.4% variance of data, respectively.

**Table 2:** Principal component analysis of socioeconomic and psychosocial variables

	**Structure matrix**	
	Factor 1	Factor 2
Perceived care from husband	0.893	
Perceived care from family	0.890	
Perceived wellbeing	0.833	
Frequency of abuse	0.584	
Affect	0.660	
Monthly family income		0.720
Education level of the husband		0.688
Use of biomass fuel		0.638

### Predictors of birthweight

Hierarchical logistic regression performed to identify factors associated with birthweight (Table 3) could predict 13.2% of the variance of birth weight (F = 6.8*, P* < *0.001*). Preterm births explained 6.9% of the unique variance in birthweight, after controlling for parity, sex of the baby, maternal age, PoG at pregnancy registration, whether having chronic/gestational diabetes, having chronic or pregnancy-induced hypertension, BMI at the time of pregnancy registration, factor score for psychosocial wellbeing and factor score for socioeconomic status. Both early pregnancy BMI and psychosocial wellbeing remained significant in the final model. According to the final model, if BMI increased by one standard deviation (4.8 kg/m^2^), the birth weight would increase by 0.21 standard deviation units (119.4 g).

**Table 3:** Hierarchal linear regression (final model) to identify factors associated with birth weight

	Beta (95% CI)	p	explained unique variance %
Parity (primy/ multi)	0.04 (-45.25, 86.11)	0.54	0.1
Sex of the baby (Male/female)	-0.08 (-163.28, 10.30)	0.08	0.7
Maternal age (years)	0.07 (-4.15, 14.83)	0.27	0.3
Gestation period at pregnancy registration (weeks)	0.03 (-11.16, 20.59)	0.56	0.1
Chronic/gestational diabetes (did not have/ Has)	0.02 (-70.73, 115.33)	0.64	0
Chronic or pregnancy-induced hypertension (did not have/had)	-0.04 (-331.59, 150.67)	0.46	0.1
BMI (kg/m^2^) at the time of pregnancy registration	0.21 (10.56, 29.61)	0	3.9
Psychosocial wellbeing	0.11 (7.47, 98.44)	0.02	1.2
Socio economic status	0.01 (-38.84, 49.03)	0.82	0
Preterm (yes/not)	0.27 (247.11, 520.31)	0	6.9

## Discussion

The current study showed that 14.6% of babies in this rural setting are born with low birthweight. Preterm birth, maternal BMI, and mid-pregnancy psychosocial wellbeing of mothers affect the birthweight of babies born in this community.

Birthweight is both an indicator and a predictor of perinatal health, and its effects are carried on further across the lifespan [8]. In Sri Lanka, the routine surveillance of public health data gives a reliable estimate of the low birthweight incidence, 11.8% in 2017 [29]. However, a detailed assessment of the distribution of birthweight or its determinants cannot be ascertained with surveillance data. Even though previous studies have shown marked geographical variations in average birthweight and low birthweight incidences at the district level, variations at micro-geographical levels are also scarce [29]. The role of the psychosocial environment of pregnant women in perinatal health in low and middle-income settings is often overshadowed by stronger determinants such as nutritional issues. Conducted in a homogenous, rural community in a middle-income country, the current study contributes to addressing these gaps in research evidence.

Marked micro-geographical variations in marital and family cohesion (including the pregnant women's perceptions regarding care received from their husbands and family members and family conflicts) have been described within this district [33]. This particular community displayed a possibly high prevalence of "abuse" compared to the rates reported in other communities. For example, 72.8% of the participants in the current study reported perceived physical, verbal, or sexual "abuse" from their husband or another household member at least once during the past month. Even though the assessment methods may vary, the previously reported prevalence of physical or sexual violence during pregnancy was 4.2% [34]. Demographic and health survey 2016 reported that.4% and 17% of ever-partnered women in Anuradhapura and Sri Lanka experienced intimate partner violence [34]. Surprisingly, despite hostile social conditions, this region has the third-lowest poverty headcount index in the district, showing that general economic status is comparatively good [31].

Stress experienced by pregnant women leads to changes in the hypothalamo-pitutary axis resulting in higher cortisol levels[35]. Higher maternal cortisol levels are associated with preterm births and lower birthweights [35]. The presence of depression or anxiety [18, 36, 37], inadequate support received from the family structure [38], experiencing violence [11], and facing stressful circumstances [19, 39] have all been associated with low birthweight or preterm births or both, emphasizing the importance of maternal psychosocial wellbeing during pregnancy. The current study has shown that the factor score for psychosocial wellbeing; the composite variable for frequency of experiencing abuse, perceived care from husband and family, perceived wellbeing, and perceived happiness during pregnancy was significantly associated with birthweight even after other possible confounders such as the economic status and medical conditions were controlled for. This highlights the importance of improving domestic cohesion, preventing violence, and improving maternal mood during pregnancy to reduce low birthweight and preterm births in this community. However, the health system will need major reorientations in the existing human and physical resources to cater to these demands [40, 41].

Maternal early pregnancy BMI is believed to be closely representative of the pre-pregnancy BMI and hence used as an indicator of the mother's nutritional status [42]. Maternal BMI was a strong predictor of birthweight in this model (4% of unique variance explained, *p* = 0.001) even after controlling for several factors, including the PoG at which the measurement was taken. However, to observe a 120 g increase in birthweight, BMI should be increased by 4.8 kg/m^2^. Though the mean birthweight of babies born to overweight and obese pregnant women was significantly higher than in other BMI categories, there was no significant difference between babies born to women with low BMI and women with normal BMI. Therefore, popular nutritional interventions aiming at pre-pregnancy BMI to prevent low birthweight will not be effective in this community.

Preterm birth was the strongest predictor of birthweight in the regression model. This was after controlling for hypertensive disorders and gestational or chronic diabetes in pregnancy which are among the commonest causes of provider-initiated preterm deliveries [43]. Therefore, why preterm births occur and how to reduce the number of preterm births need to be investigated.

Proxy indicators used during field care provision to monitor fetal growth cannot effectively predict birthweight. Total weight gain during pregnancy showed a very weak correlation with birthweight (*r* = 0.094, *P 0.038*). Third-trimester SFH being compatible with the PoG and having gained weight according to national recommendations were not shown to be significantly associated with the birthweight.

### Strengths and limitations

The entire study population was invited to participate in the study. Loss to follow-up was 6.5%. The proportion of births with low birthweight in the study sample was higher than the national average (14.6%, 95% CI 11.7–17.9). However, the percentage of births with low birthweight in the Ipalogama MOH area in 2017 (routine surveillance data) was 15.9%, which is within the 95% confidence limits estimated from the sample.

All the deliveries in the cohort occurred in government hospitals. Therefore, the birthweight had been assessed and recorded following the same national guideline for birthweight measurement. Calibrated equipment was used. Outcome data were extracted from these medical records, which ensures higher data quality. Secondary data extracted from the pregnancy record were used as the mother's demographic characteristics, obstetric and medical history, anthropometric measurements, hemoglobin level, and plasma glucose values. Even though the maintenance of health records was standardized and monitored, some heterogeneity of these data could be expected.

Anuradhapura is the largest geographical district in the country, and heterogeneity in socio-cultural aspects is evident between subpopulations of the district [33, 44]. Therefore, looking at the epidemiology of perinatal outcomes at microgeographic levels is important to quantify the effect of such factors. Since this study was conducted in a homogenous community, socio-cultural factors' association with birthweight was brought into prominence.

The cohort was defined as women whose index pregnancy was continued at least up to 28 weeks of gestation. Therefore, the findings may not apply to extreme preterm deliveries.

The outcome variable 'birthweight' has many known and unknown predictors and confounders. The predictor variables used in the regression model are limited, and as expected, a low percentage (13.2%) of the variability could be explained using these variables. Other major determinants of birth weight should always be considered in planning interventions for improving birth weight.

## Conclusions

In a setting where a comparatively higher proportion of pregnant women suffer 'abuse' in their homes, psychosocial wellbeing during pregnancy, a largely neglected aspect in routine maternal care provision, was an important determinant of birthweight of babies. Strengthening the health system approach beyond routine nutrition-focused interventions to address the social determinants and psychological wellbeing in pregnant women would help improve perinatal health in developing countries.

## Data Availability

The datasets used and analyzed during the current study are available from the corresponding author on reasonable request.

## Abbreviations

ANOVA: Analysis of Variance
BMI: Body Mass Index
CHDR: Child Health and Development Record
MOH: Medical Officer of Health
PoG: Period of Gestation
PPBS: Post-Prandial Blood Sugar
SFH: Symphysio-Fundal Height
